# Advancing Understanding of High-Temperature Micro-Electro-Mechanical System Failures with New Simulation-Assisted Approach

**DOI:** 10.3390/s25103120

**Published:** 2025-05-15

**Authors:** Weronika Lidia Sadurska, Matthias Imboden, Jürgen Burger, Alex Jean Dommann

**Affiliations:** 1 School of Biomedical and Precision Engineering, University of Bern, 3012 Bern, Switzerland; weronikasadurska@gmail.com (W.L.S.); juergen.burger2@unibe.ch (J.B.); 2 4K-MEMS S.A., 2072 St-Blaise, Switzerland; 3 ARTORG Center for Biomedical Engineering Research, University of Bern, 3012 Bern, Switzerland

**Keywords:** high-temperature MEMS, failure mechanism, degradation, simulations, atomic migration, tungsten

## Abstract

High-temperature micro-electro-mechanical systems (MEMSs) are critical for applications in extreme environments and applications where the operating temperature can exceed 1000 °C, but their long-term performance is limited by complex failure mechanisms, including material degradation caused by atomic migration. This study introduces a simulation-assisted approach to analyze and predict the dominant failure modes, focusing on vacancy fluxes and their driving forces, within high-temperature MEMS structures. The focus is on tungsten-based structures operating at a temperature of 1580 °C. This approach couples electric-, stress- and temperature-dependent simulations to evaluate atomic migration pathways, which are key contributors to failure. This study demonstrates that void accumulation, driven by vacancy migration, results in localized current density increase, hotspot formation, and accelerated structural degradation. The mean time to failure (MTTF) is shown to have exponential dependence on temperature and inverse-square dependence on current density, highlighting the critical role of these parameters in device reliability. These findings provide a deeper understanding of the failure mechanisms in high-temperature MEMSs and underscore the need for design strategies that mitigate electromigration and stress-induced void growth to enhance device performance and longevity.

## 1. Introduction

Microelectromechanical systems (MEMSs) have revolutionized various industries with their compact size, efficiency, and versatility, particularly in low-temperature applications. However, as MEMS technology advances, there is a growing demand for devices capable of operating in extreme and harsh environments, including temperatures exceeding 1000 °C. These high-temperature MEMS structures are crucial for applications in aerospace [1] and automotive sensors [2], thermal infrared (IR) sources [3], or chemical sensing microsystems [3,4], where low-temperature MEMS devices fail to perform due to their limited reliability in higher temperature ranges.

### 1.1. High-Temperature MEMS Performance

The performance and reliability of MEMS devices in high-temperature environments differ significantly to those operating at lower temperatures. Most of the MEMS structures, which are used for higher-temperature applications, such as microhotplates (MHPs), have operating temperatures up to 1000 °C. Several examples of such structures can be found in the literature, with heater materials including platinum [5,6,7], polysilicon [8], titanium nitride [9], or tungsten [10,11,12,13]. Some limited examples of MEMS structures operating above 1000 °C can be found, where the heater material is platinum [14], molybdenum [15], or boron-doped polycrystalline diamond PCD [16]. Failure mechanisms with corresponding failure temperatures for different heater materials used as microhotplates are shown in Table 1.

The operating temperature significantly impacts the performance of these devices, making stability and failure mechanisms at high temperatures crucial for MHPs. In accelerated life testing, the Arrhenius relationship is commonly used to model how temperature influences device reliability over time. The atomic migration failure can be described by this relationship. This failure mechanism is strongly temperature-dependent as the atomic mobility increases with the temperature and so does diffusivity [19]. The failure caused by hotspot formation is also significantly influenced by Arrhenius kinetics as the higher temperatures can cause larger temperature gradients, leading to failure [5,17]. The Arrhenius relationship shows that the mean time to failure (MTTF) has an exponential temperature dependence, expressed by the following equation [19]:

(1) $$MTTF=A_{0}\exp\left(\frac{E_{a}}{kT}\right)$$

In this formula, *A*_0_ is a pre-exponential factor that varies depending on the material properties, device design, and environmental factors of the system being analyzed, $E_{a}$ is the activation energy related to the failure mechanism, and *k* is the Boltzmann constant. Even small increases in temperature *T* can greatly reduce the lifespan of a device as higher temperatures significantly accelerate failure mechanisms [19]. This exponential temperature dependence makes it essential to study failure modes at elevated temperatures in order to improve the reliability of high-temperature devices.

Failure mechanisms such as electromigration [6,7,13], hotspot formation [5,18], the rupture of the membrane [9,11,15], and the displacement of the suspended structure [18] have been observed in MEMS structures operating at temperatures up to 1000 °C.

Despite the increasing interest in high-temperature MEMSs, the current literature lacks a comprehensive discussion on the specific failure mechanisms that occur in devices operating above 1000 °C. Much of the existing research on MEMS reliability focuses on low- to moderate-temperature environments, leaving a gap in understanding how high temperatures influence failure modes and overall performance. This study addresses this gap by presenting a novel approach to analyzing the failure modes of high-temperature thermal MEMSs through a combination of experimental observations and simulation-assisted investigations. The aim is to enhance the understanding of how extreme temperatures affect MEMS reliability, ultimately improving the performance and longevity of these critical systems.

### 1.2. Atomic Migration as a Failure Mechanism

Among the potential failure mechanisms at temperatures higher than 1000 °C, atomic migration phenomena is crucial. Atomic migration refers to the movement of atoms within a material, often driven by external forces like temperature gradients, stress, or electric fields. The following types of atomic migration can be distinguished: electromigration (driven by an electric field), thermomigration (driven by temperature gradients), and stress-induced migration (driven by hydrostatic stress gradients) [19,20,21]. These migration processes can lead to void formation or material accumulation, leading to open-circuit or structural failure, especially in metallic components such as metallic interconnects or heating elements commonly used in thermal MEMSs [19,21,22]. Prior studies at lower temperatures have shown that such atomic migration mechanisms significantly influence device reliability [6,7,13], but their behavior in MEMS devices operating at *T* > 1000 °C remains mostly underexplored. Adapting and transferring models of failure modes observed in low-temperature MEMSs to high-temperature MEMSs operating at *T* > 1000 °C, like atomic migration caused by an electric field, hydrostatic stress gradient and temperature gradient, can potentially show that these failure modes are also relevant in high-temperature tungsten MEMSs. Such high-temperature MEMS devices can be specifically designed for use as IR sources. These type of applications inherently require significantly elevated operating temperatures in order to achieve thermal emission in the infrared spectral range. According to Planck’s law, emission in the mid- to long-wave IR region demands high blackbody temperatures, which places constraints on material selection, device design, and reliability [3,16].

## 2. Materials and Methods

The models simulated in this work are based on tungsten MEMS hotplates that are fabricated using standard MEMS micromachining methods. The exact process flow and design are proprietary, and the concepts are disclosed in patents [23,24].

### 2.1. Tungsten MEMS Hotplates

**Figure 1:**
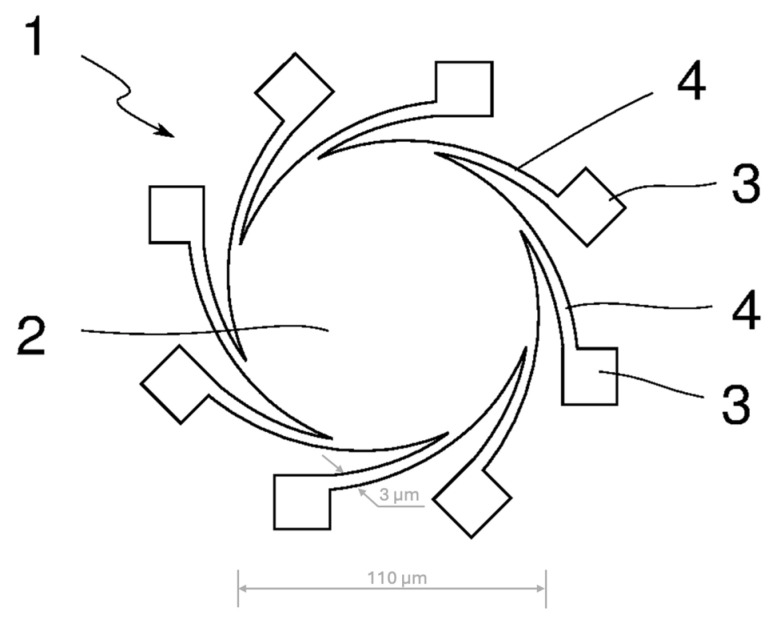
A simplified schematic of the studied tungsten microhotplates (the top view of the structure). The numbered labels correspond to components as defined in the original patent: 1—radiator device, 2—emitter plate, 3—electric pads, 4—heater arms [24].

The MEMS hotplates are fabricated out of a single material, in this case, sputtered tungsten, such that the central hotplate and heater arms form a single structure. Optical lithography is used to pattern the precise shape of the heater elements. The hotplates are suspended above the substrate by a plurality of heaters, as shown in Figure 1. The simplified schematic shows the top view of a tungsten microplate (reproduced from the patent US20230131181A1). The emitting plate has an area of 0.02 ${mm}^{2}$. The arm length may vary; in this case, it is 80 µm, and the thickness is 2 µm. The width of the narrowest part of the arms is 3 µm by design but may vary after the fabrication process is complete [24]. The dimensions of the hotplate and heater arms studied in this article are marked in Figure 1. The devices are operated in vacuum, estimated to be <10^−5^ mbar, such that there are no chemical interactions between an atmosphere and the hotplates, important for longer operations. Even a short operation is not possible in the presence of a gas, including inert gases, as the devices would be thermally shorted to the package.

The devices are heated by applying a voltage in the range of 0.5–0.8 V (either DC or AC) to the electric pads, which heat the central plate to temperatures in excess of 1700 °C. The temperature is measured using a visible infrared (VIS/IR) spectrometer by fitting the emission intensity between 400 nm and 950 nm to Plank’s equation. The clamps remain cool, close to ambient temperature, while the center plate reaches a high, uniform temperature, close to the maximum temperature. Thermal pathways are limited to conduction through the material and radiative cooling, which starts to become significant above 1700 °C (typically, radiative power is on the order 5–15% of the total power, depending on the exact design). The radiative cooling of the center means that the hottest point of the emitters is on the heaters, close to the attachment point to the hotplate.

**Figure 2:**
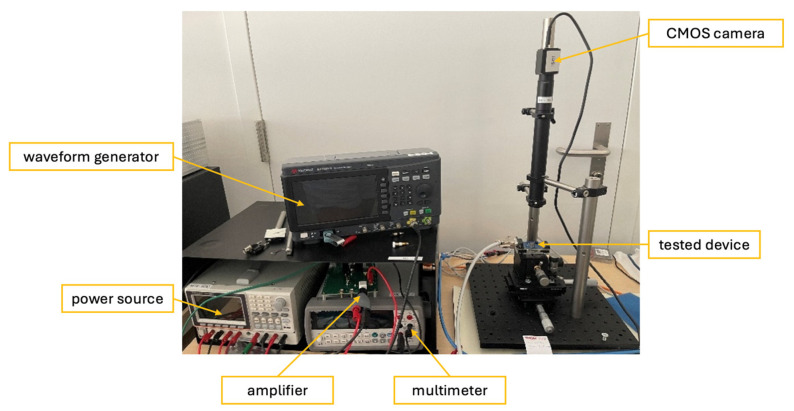
Testing bench.

The emitters were tested using a power source (GW Instek GPP-4323, China), amplifier (Texas Instruments OPA548EVM, TX, USA), waveform generator (Keysight 33220A, CA, USA), and a multimeter (Keysight 34460A, CA, USA). A CMOS camera (IDS Imaging Industrial camera UI-6240SE-M-G, Germany) was used for capturing live images during operation and used to track how the device aged. The setup is presented in Figure 2.

**Figure 3:**
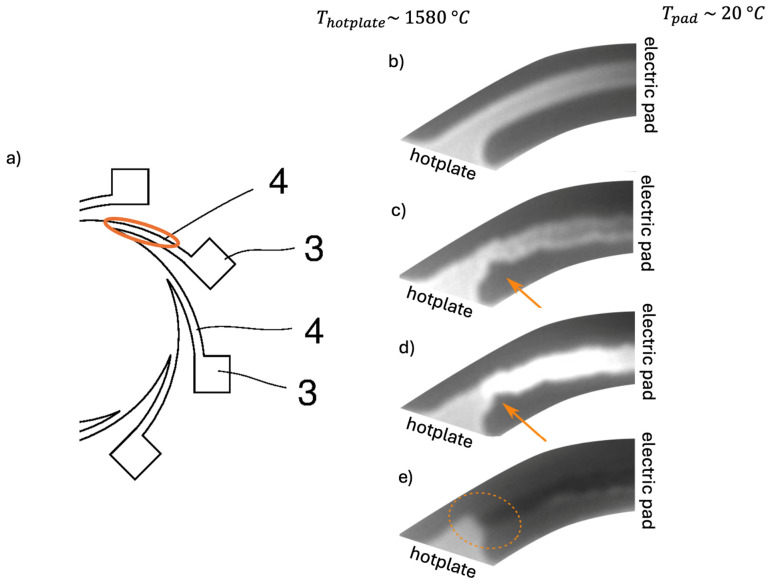
Tungsten microhotplate. (**a**) Cross-section of schematic with marked area of focus; (**b**) image of heater arm at start of test at 1580 °C; (**c**) image of heater arm after 224 h, just before failure; (**d**) image of heater arm failing due to visibly increased current density; (**e**) image of heater arm after device failure: fracture on heater.

Figure 3 illustrates the hottest areas of the emitters located close to the attachment point of the hotplate. Figure 3a shows a cross-section of the schematic of the studied microhotplates, with the hottest area indicated by the orange-colored arrow. Figure 3b–e show the aging of the heater elements. The testing conditions of the device shown in Figure 3 can be found in Table 2.

The images depicted in Figure 3 are taken at a wavelength of 900 nm, where the light observed is emitted from the device itself. Figure 3b shows a pristine device operating at 1577 °C. Figure 3c is an image of the same heater after 224 h. Notching, or void formation, is clearly visible, a result of atomic migration driven by electromigration, stress gradients or concentration gradients.

The purpose of this work is to quantify the different atomic migration drivers and simulate which failure mechanism dominates. Figure 3d,e illustrate the heaters after failure. In Figure 3e, a break is visible, resulting in a colder (darker) heater. The hotplate is still hot as the remaining heater elements are still functional and in part hotter than before due to the increase in current Figure 3d.

### 2.2. Vacancy Flux Model

Atomic migration is a term encompassing various processes of material transport within solid materials. In metals, atomic migration typically occurs through the movement of mobile defects, such as vacancies. This migration process is governed by diffusion laws, where atoms move by occupying neighboring vacancies. Fick’s first law of diffusion relates diffusive flux *J* to the gradient of concentration *c*. It suggests that the movement of particles from high to low concentration is directly proportional to the concertation gradient [25]:

(2) $$J=-D\frac{\partial c}{\partial x}$$

The number of atoms diffusing per unit time across a unit area per unit concentration gradient is known as diffusivity or diffusion coefficient *D*.

In metals, diffusion typically follows the vacancy diffusion mechanism. Atoms move from one lattice site to another, provided they have sufficient energy to overcome a local activation energy barrier and that vacancies are available. The activation energy for diffusion is the sum of the energy required to form a vacancy and the energy needed to move an atom into the vacancy. In addition to bulk diffusion, grain boundary diffusion plays a crucial role in metals. Here, net mass transport occurs along grain boundaries, which may act as sources or sinks for atomic fluxes. Grain boundary diffusion, which occurs through a vacancy mechanism, is significant across both small-angle and large-angle boundaries. Diffusivity for vacancy diffusion along the grain boundaries can be described by the Arrhenius law [20]:

(3) $$D=D_{0}\exp\left(\frac{-E_{aDb}+\sigma_{hyd}\Omega }{kT}\right)$$

where $D_{0}$ is the pre-exponential factor and $E_{aDb}$ is the activation energy for the diffusion along the grain boundaries. Hydrostatic stress $\sigma_{hyd}$ and atomic volume Ω take into account the effect of mechanical stress on diffusion [20].

In metals, atomic migration involves vacancy diffusion, where material transport is expressed in terms of vacancy flux ${\vec{J}}_{v}$. When the vacancy flux diverges, vacancies can accumulate or diminish based on the divergence’s sign, as governed by the continuity equation:

(4) $$\frac{\partial c_{v}}{\partial t}=-\nabla {\vec{J}}_{v}+G$$

where $c_{v}$ is the vacancy concentration and *t* corresponds to time.

The source term *G* accounts for vacancy generation or annihilation and depends on vacancy relaxation time *τ* relative to equilibrium vacancy concentration $c_{veq}$:

(5) $$G=\frac{c_{v}-c_{veq}}{\tau }=\frac{c_{v}-c_{v0}\exp\left(\frac{\left(1-f\right)\sigma_{hyd}\Omega }{kT}\right)}{\tau }$$

where relaxation time *τ* determines the rate at which vacancy concentration reaches a steady state, with smaller *τ* values corresponding to faster stabilization, and *f* is a relaxation factor. The initial vacancy concentration, $c_{v0}$, is defined as

(6) $$c_{v0}=N\;exp\left(\frac{-E_{av}}{kT}\right)$$

where *N* is a total number of atomic sites and $E_{av}$ is the vacancy formation energy.

In metals, atomic migration, primarily through vacancy diffusion, contributes to total material transport and can be described in terms of vacancy flux, incorporating vacancy fluxes driven by external forces. The driving forces for atomic migration in microstructures like hotplates are a function of the electric field, $\vec{E}$, hydrostatic stress gradient, $\nabla \sigma_{hyd}$, and/or temperature gradient, ∇*T*. Vacancy migration is a specific type of atomic migration where an atom moves to a nearby vacancy. As the atoms move, the vacancy moves in the opposite direction.

The following vacancy fluxes can be distinguished due to various driving forces [20]:

- A vacancy flux due to the electric field:

(7) $$PFlux=\frac{\left|z^{*}\right|e}{kT}Dc_{v}\vec{E}$$

where z* is the effective charge number and *e* is the elementary charge.

- A vacancy flux due to the temperature gradient:

(8) $$TFlux=\frac{Q^{*}}{kT^{2}}Dc_{v}\nabla T$$

where Q* is the heat of transport.

- A vacancy flux due to the hydrostatic stress gradient:

(9) $$SFlux=-\frac{f\Omega }{kT}Dc_{v}\nabla \sigma_{hyd}$$

The total vacancy flux is present in the form [20]

(10) $${\vec{J}}_{v}=-D\nabla c_{v}+\frac{\left|z^{*}\right|e}{kT}Dc_{v}\vec{E}+\frac{Q^{*}}{kT^{2}}Dc_{v}\nabla T-\frac{f\Omega }{kT}Dc_{v}\nabla \sigma_{hyd}$$

In this equation, the first term describes a diffusion effect and results in a flow from high to low vacancy concentration, while the other terms represent driven fluxes: the second term represents electromigration, the third term represents the material transport, which occurs due to thermal gradients, and the last term accounts for the atomic migration caused by gradients of hydrostatic stress [20]. The driving force behind atomic migration-induced failure stems from the accumulation of vacancies leading to void formation and the accumulation of atoms leading to hillock formation [22]. Hillocks can grow, causing short-circuits, while vacancies can grow and eventually result in open circuits [21].

### 2.3. Simulation Model Development

The simulation model developed here is based on a tungsten microhotplate suspended above the substrate by six heaters, described in Section 2.1., with dimensions shown in Figure 1. The simulation model was created in COMSOL (version 6.2) using the following modules:

- Solid Mechanics;
- Electric Currents;
- Heat Transfer in Solids;
- Multiphysics;
- Transport in Solids.

The first four modules were used to accurately simulate the electro thermomechanical behavior of the system, including the resistive heating of the device, thermal expansion due to heating, conduction through the material, the radiative cooling of the plate, and mechanical fixed constraints at the clamps. The Transport in Solids module was used to introduce mass transport that is specified by Fick’s law of diffusion. Vacancy generation and annihilation are controlled by the source term, and the vacancy fluxes are introduced by external flux nodes. As for the boundary conditions, the conditions of the simulated environment are presented in Table 3. The ambient conditions were set at room temperature. The voltage was applied to half of the electric pads and was set to 0.59 V (with the remaining pads held at ground) to reproduce the testing conditions and to reach a temperature of approximately 1580 °C of the device, shown in Figure 3. The mechanical fixed constraint was placed at all six clamps. Thermal pathways were limited to conduction through the material and radiative cooling.

A list of the input parameters, their values and references can be found in Table 4.

A list of the variables related to atomic migration and derived values from the simulation are shown in Table 5. For all formulas presented in this table, a complete list of the symbols and their definitions are provided in the Nomenclature section.

The following material was selected and applied to whole geometry from the COMSOL Material Library:

Material Library > Elements > Tungsten > Phase: solid > Variation: Binkele.

With this choice of material, all material properties required from the simulations are specified in the material library as being temperature-dependent.

To create a mesh that is suitable for simulating the system, an extra-fine mesh element size was chosen as the variables listed in Table 5 were dependent on hydrostatic stress, which requires a fine mesh to simulate accurately. The stress simulations are sensitive to the mesh size, and applying a sufficiently fine mesh is critical to ensure an accurate simulation of the terms related to stress. To validate the accuracy of the simulation, a mesh convergence study was performed, confirming that the chosen mesh resolution captured the stress gradients without numerical artifacts.

## 3. Results

### Simulation Results

**Figure 4:**
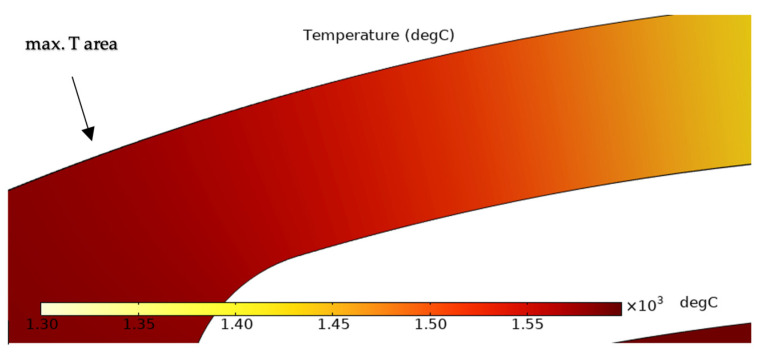
Simulated temperature distribution.

**Figure 5:**
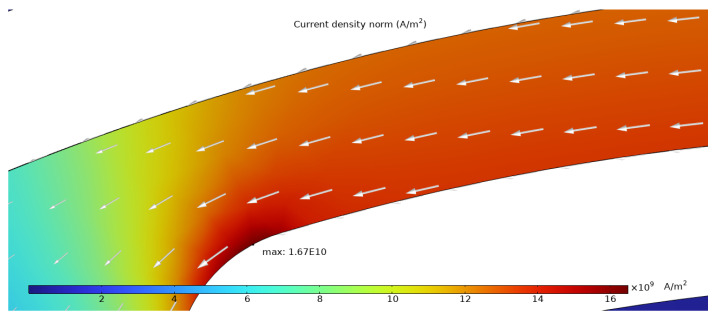
The simulated current density distribution with white arrows marking the direction of the current.

**Figure 6:**
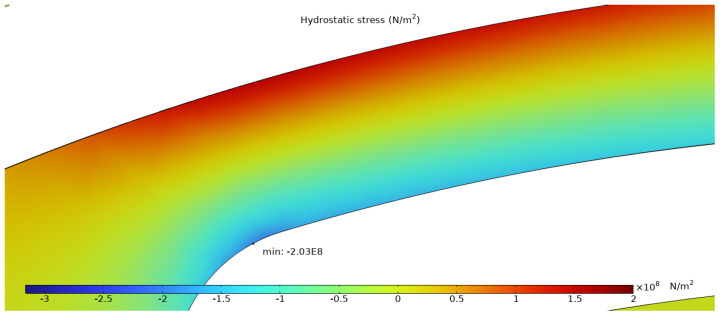
Simulated distribution of hydrostatic stress.

**Figure 7:**
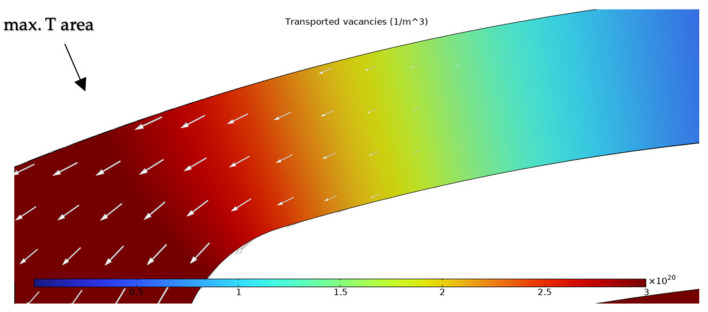
The simulation results of the transported vacancy concentration with white arrows marking the vacancy transport vector.

**Figure 8:**
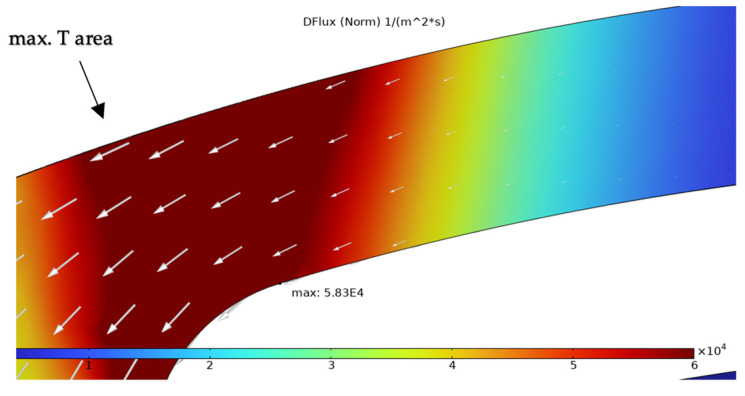
Simulation result of vacancy flux due to diffusion (*DFlux*) with white arrows marking vacancy transport vector.

**Figure 9:**
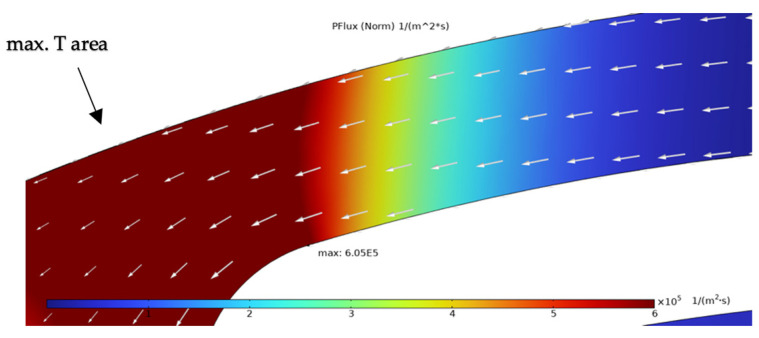
Simulation result of vacancy flux due to electric field (*PFlux*) with white arrows marking vacancy transport vector.

**Figure 10:**
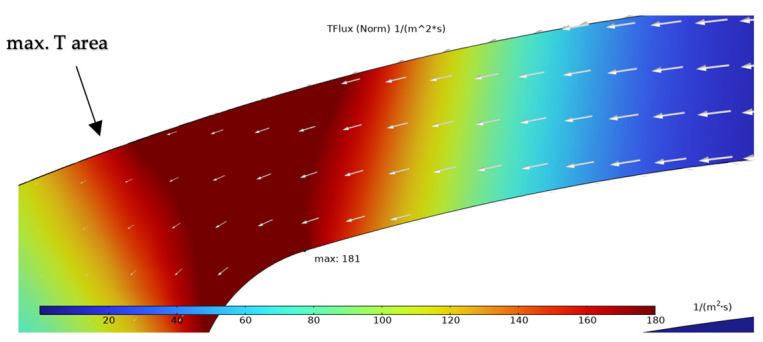
Simulation result of vacancy flux due to temperature gradient (*TFlux*) with white arrows marking vacancy transport vector.

**Figure 11:**
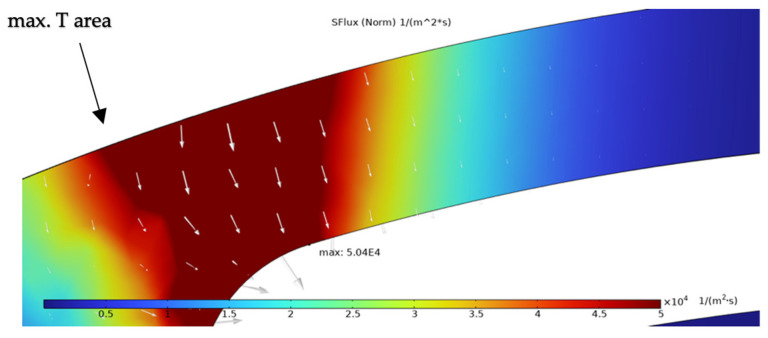
Simulation result of vacancy flux due to hydrostatic stress gradient *(SFlux)* with white arrows marking vacancy transport vector.

**Figure 12:**
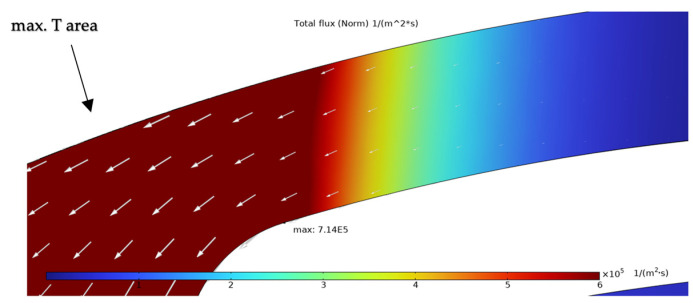
Simulation result of total vacancy flux with vacancy transport indicated by arrows.

The simulation results presented in this chapter are limited to the failure area of the device. The following outputs were generated from the steady-state simulations: maximum temperature, maximum current density norm, average current density, maximum hydrostatic stress (Table 6), temperature distribution (Figure 4), current density distribution (Figure 5), hydrostatic stress distribution (Figure 6), maximum values of the vacancy fluxes leading to atomic migration (Table 7), the distribution of the transported vacancy concentration (Figure 7), vacancy fluxes due to their driving forces and total vacancy flux (Figure 8, Figure 9, Figure 10, Figure 11 and Figure 12).

The maximum value of the temperature exceeds 1500 °C, and as for the experimental results shown in Figure 4, the hottest part is located close to the hotplate. There is a temperature gradient visible along the heater arm.

The current density distribution, presented in Figure 5, exhibits high current density at the right side of the structure and shows current concentration at the curved portion of the structure with a maximum of $1.67\times 10^{10}\frac{A}{m^{2}}$. The heater arm has an average current density of $1.34\times 10^{10}\frac{A}{m^{2}}$.

The hydrostatic stress distribution, shown in Figure 6, illustrates the presence of a stress gradient (both positive and negative values of hydrostatic stress are present). The absolute maximum value is located on the curved portion of the structure and has a value of $2.03\times 10^{8}$ $\frac{N}{m^{2}}$. The negative hydrostatic stress values are located at the bottom of the arm (compression), whereas the positive values are located at the top of the structure (tension).

The simulation results of vacancy fluxes and their percentage of the total vacancy flux are summarized in Table 7.

The transported vacancy concentration is shown in Figure 7. The transported vacancy concentration is the total vacancy concentration times the fraction of vacancies that are mobile. The arrows indicate the movement of the vacancies. The highest vacancy concertation is located in the area of maximum temperature in the left portion of the heater arm.

The distribution of the vacancy flux due to diffusion (*DFlux*) is shown in Figure 8. The arrows mark the direction amplitude of vacancy transport caused by diffusion. The maximum of this flux is located in the area of the highest temperature. The diffusive flux contributes to the total vacancy flux by 8.17%.

The vacancy flux due to the electric field and its distribution is shown in Figure 9. The maximum of this flux is concentrated in the left portion of the structure in the area of the highest temperature and the point of the highest current density. The arrows indicate vacancy transport related to this flux mechanism. *PFlux* contributes to the total flux by 84.73%.

Figure 10 presents the distribution of the vacancy flux due to the temperature gradient with its concentration located at the place of the existing temperature gradient after the maximum temperature area. *TFlux* contributes 0.03% to the total vacancy flux, with the directions marked by white arrows.

The vacancy flux due to hydrostatic stress gradients and their distribution is shown in Figure 11 with a maximum located at the left side of the structure close to the highest temperature area. The arrows mark the vacancy transport direction related to *SFlux,* which contributes to the total vacancy flux by 7.06%.

The total vacancy flux with marked vacancy transport vectors is shown in Figure 12. The maximum value of this flux is located at the left side of the heater arm with the maximum value at the curved part.

## 4. Discussion

The simulation results show that the region of the highest temperature (Figure 4), current density (Figure 5) and hydrostatic stress (Figure 6) is common in the structure considered, meaning that high values of these parameters are all present in approximately the same region, leading to the accumulation of the effects. This can also be seen in terms of the distribution of vacancy fluxes shown from Figure 8, Figure 9, Figure 10 and Figure 11. The regions with the highest values of vacancy are in almost the same location, leading to the total vacancy flux, shown in Figure 12, to be concentrated at the left side of the structure in the curved attachment area. The transported vacancy concentration presented in Figure 7 shows that the accumulation of vacancies is present at the place where the failure of the experimental device occurs, as shown in Figure 3.

The simulation results of vacancy fluxes in Table 7 summarize that vacancy flux is dominated by electromigration. The second largest contribution to vacancy flux is due to diffusion and then, at a very similar magnitude, the flux is due to the hydrostatic stress gradient. Their influence can be treated as equally important. The flux that has the smallest contribution is caused by the temperature gradient and its influence is approximately four thousand times smaller compared to the flux due to the electric field. Based on these results, real-life device failure is most likely driven by electromigration resulting from high current densities, resulting in the driving force of atomic migration, as the visible material transport in Figure 3 originates at the area proven most affected by the simulated flux migration. Additionally, Figure 11 shows the vacancy flux due to the hydrostatic stress gradient, with the vacancies moving from the positive regions of the hydrostatic stress to the negative regions. This corresponds to the notching effect observed in the heater arm in Figure 3c,d, indicating that vacancies accumulate at the region of negative hydrostatic stress, leading to void formation.

Besides the importance of the main driving forces in the formula for total vacancy flux (Equation (10)), it can be seen that the magnitude of each term is dependent on diffusivity and vacancy concentration. In terms of those two terms, we can see in the formulas presented in Table 5 that they are dependent on temperature and hydrostatic stress. Vacancy formation is facilitated when hydrostatic stress is tensile compared to when it is compressive, and high temperature is also a favorable factor for vacancy creation as thermal energy is needed to overcome energy gap *E_A_*. That means that temperature and hydrostatic stress are crucial parameters related to atomic migration.

Void growth is the most concerning failure case in the described system as void creation and growth will locally increase the current density, leading to hotspot formation and stress increase, causing the further expansion of the voids, as presented in Figure 3d, and this leads to further hotspot formation that causes the ultimate device failure (Figure 3e). The uneven stress distribution caused by the voids exacerbates mechanical failure, weakening the structural integrity of the heater arm.

Electromigration is shown to be the leading cause of failure in the tested and simulated tungsten microhotplates. To relate the effect of atomic migration caused by high current density, the equation of MTTF (Equation (1)) can be transformed to include current density *j* [20] as follows:

(11) $$MTTF=\frac{A_{0}}{j^{2}}exp\left(\frac{E_{a}}{kT}\right)$$

The MTTF has, in this case, not only exponential dependence on the temperature but also inverse-square dependence on the current density. This shows that the lifetime and reliability of the device will decrease significantly in the presence of high temperature and high current density.

## 5. Summary

This study explores the dominant failure mechanisms in high-temperature MEMS devices with a heater element made of tungsten, focusing on the combined effects of temperature, current density, and hydrostatic stress on atomic migration. Steady-state simulations were performed to extract critical parameters, including temperature distribution, current density, hydrostatic stress, and vacancy flux contributions caused by diffusion, electric fields, temperature gradients, and stress gradients.

The results highlight that the regions of maximum temperature, current density, and hydrostatic stress coincide in the structure, particularly at the curved portion of the heater arm near the hotplate. This overlap leads to concentrated vacancy fluxes and accumulated vacancy transport, correlating with real-life device failures where void formation is observed. Among the driving forces for atomic migration, electromigration (flux due to the electric field) was found to dominate, contributing 84.73% to the total vacancy flux. Vacancy fluxes caused by diffusion (8.17%) and hydrostatic stress gradients (7.06%) also play significant roles, whereas the temperature gradient had a negligible contribution to the driving force of the vacancies (0.03%).

The simulations further revealed that vacancies migrate from regions of positive hydrostatic stress to regions of negative stress, causing void accumulation. This notching effect aligns with physical observations of failure sites and the subsequent hotspot formation in heater arms. Void growth accelerates local current density, leading to a positive feedback loop of increased stress, further void expansion, and eventual structural failure.

This study underscores the critical role of temperature and current density in device reliability, as reflected in the mean time to failure (MTTF) equation. MTTF has exponential dependence on temperature and inverse-square dependence on current density, illustrating how extreme conditions significantly shorten the operational lifetime of high-temperature MEMS devices. This work provides valuable insights into the underlying failure mechanisms and emphasizes the importance of mitigating electromigration and stress-driven degradation to improve MEMS device robustness and longevity.

## Figures and Tables

**Table 1:** Failure temperatures and failure types for different heater materials used as microhotplates.

Heater Material	Temperature Reached at the Time of Failure	Failure Mechanism	Reference
Pt	870 °C	Hotspot formation	[5]
Pt	700 °C	Electromigration	[7]
W	750 °C	Rupture of membrane	[11]
Pt	820 °C	Electromigration	[6]
TiN	720 °C	Rupture of membrane	[9]
Al	350 °C	Hotspot formation	[17]
Pt	550 °C	Displacement of suspended structures	[18]
W	850 °C	Electromigration and stress migration	[13]
Pt	1057 °C	Thermomigration	[14]
Mo	1360 °C	Rupture of membrane	[15]

**Table 2:** Testing conditions.

Testing Specifications	
Voltage	0.6 V
Current type	DC
Hotplate temperature	1577 °C

**Table 3:** Conditions of the simulated environment.

Environmental Conditions	Value	Units
Ambient temperature $T_{0}$	20	°C
Reference temperature $T_{ref}$	20	°C
Applied voltage $V_{0}$	0.59	V

**Table 4:** A list of parameters specified for the simulation of atomic migration in a tungsten high-temperature MEMS.

Parameter	Value	Units	Reference
Pre-exponential factor $D_{0}$	${5.2\times 10}^{-8}$	$\frac{m^{2}}{s}$	[26]
Diffusion activation energy $E_{aDb}$	5.2	eV	[25,27]
Vacancy formation activation energy $E_{av}$	3.3	eV	[25,28,29]
Molar mass of tungsten $A_{W}$	183.84	$\frac{g}{mol}$	[30]
Vacancy relaxation ratio *f*	0.73	-	[31]
Vacancy relaxation time τ	$10^{-4}$	s	[32]
Atomic volume Ω	$2.48^{-29}$	$\frac{m^{3}}{atom}$	[27]
Effective valence $z^{*}$	−20	-	[27]
Heat of transport $Q^{*}$	${9.4\times 10}^{-3}$	eV	[27]

**Table 5:** List of variables calculated during simulation.

Parameter	Equation	Unit	References
Diffusivity	$D=D_{0}\exp\left(\frac{-E_{aD}+\sigma_{hyd}\Omega }{kT}\right)$	$\frac{m^{2}}{s}$	[19,20,25]
Initial vacancy concentration	$c_{v0}=Nexp\left(\frac{-E_{av}}{k*T}\right)$	$\frac{1}{m^{3}}$	[25]
Total number of atomic sites	$N=\rho \;\frac{N_{A}}{M}$	$\frac{1}{m^{3}}$	[25]
Equilibrium vacancy concentration	$c_{veq}=c_{v0}exp\left(\frac{\left(1-f\right)\sigma_{hyd}\Omega }{kT}\right)$	$\frac{1}{m^{3}}$	[20,33]
Source term	$G=-\frac{c_{v}-c_{veq}}{\tau }$	$\frac{1}{m^{3}s}$	[20,33]
Vacancy flux due to diffusion	*DFlux =* $-D\nabla c_{v}$	$\frac{1}{m^{2}s}$	[19,20,27]
Vacancy flux due to electric field	$PFlux=\frac{\left|z^{*}\right|e}{kT}Dc_{v}\vec{E}$	$\frac{1}{m^{2}s}$	[19,20,27]
Vacancy flux due to temperature gradient	$TFlux=\frac{Q^{*}}{kT^{2}}Dc_{v}\nabla T$	$\frac{1}{m^{2}s}$	[19,20,27]
Vacancy flux due to stress gradient	$SFlux=-\frac{f\Omega }{kT}Dc_{v}\nabla \sigma_{hyd}$	$\frac{1}{m^{2}s}$	[19,20,27]

**Table 6:** Simulation results of maximum temperature, maximum and average current density norm and maximum hydrostatic stress values.

Temperature [°C]	Current Density Norm [A/m^2^]	Average Current Density [A/m^2^]	Hydrostatic Stress (Absolute) [N/m^2^]
1580	$1.67\times 10^{10}$	$1.34\times 10^{10}$	$2.03\times 10^{8}$

**Table 7:** The simulation results of the vacancy fluxes and the total vacancy flux—maximum values.

	*DFlux (Norm)* [1/(m^2^s)]	*PFlux (Norm)* [1/(m^2^s)]	*TFlux (Norm)* [1/(m^2^s)]	*SFlux (Norm)* [1/(m^2^s)]	*Total Flux (Norm)* [1/(m^2^s)]
Results	$5.83\times 10^{4}$	$6.05\times 10^{5}$	$1.81\times 10^{2}$	$5.04\times 10^{4}$	$7.14\times 10^{5}$
Normalized results	8.17%	84.73%	0.03%	7.06%	100%

## Data Availability

Data are contained within the article.

## Nomenclature

*D*	Diffusivity
$D_{0}$	Pre-exponential factor
$E_{aDb}$	Grain boundary diffusion activation energy
$E_{av}$	Vacancy formation energy
$\vec{E}$	Electric field
*G*	Generation or annihilation source
${\vec{J}}_{v}$	Total vacancy flux
*M*	Molar mass
*N*	Total number of atomic sites
$N_{A}$	Avogadro constant
*Q**	Heat of transport
*T*	Temperature
*V*	Voltage
*Z**	Effective charge number
*c*	Concentration
$c_{v}$	Vacancy concentration
$c_{v0}$	Initial vacancy concentration
$c_{veq}$	Equilibrium vacancy concentration
*e*	Elementary charge
*f*	Vacancy relaxation factor
*k*	Boltzmann constant
*x*	Coordinate
*t*	Time
*Ω*	Atomic volume
*ρ*	Mass density
$\sigma_{hyd}$	Hydrostatic stress
$\sigma_{Mises}$	Von Mises stress
τ	Vacancy relaxation time
